# Prevalence of obstructive sleep apnea in patients with peripheral arterial diseases

**DOI:** 10.1007/s11325-019-01950-z

**Published:** 2019-11-14

**Authors:** Filip M. Szymanski, Dariusz Gorko, Anna E. Platek, Tomasz Ostrowski, Krzysztof Celejewski, Witold Chudzinski, Anna Szymanska, Kamil Stepkowski, Anna Rys-Czaporowska, Karolina Semczuk-Kaczmarek, Zbigniew Galazka, Krzysztof J. Filipiak

**Affiliations:** 1 Department of Cardiology, Medical University of Warsaw, Warsaw, Poland; 2 Department of General and Endocrinological Surgery, Medical University of Warsaw, Warsaw, Poland; 3 Cardiology Department, Regional Hospital, Kolobrzeg, Poland; 4 Department of General and Experimental Pathology, Medical University of Warsaw, Warsaw, Poland; 5 Department of Heart Diseases, Medical Centre of Postgraduate Education, Warsaw, Poland

**Keywords:** Peripheral arterial diseases, Obstructive sleep apnea, Atherosclerosis, Epidemiology

## Abstract

**Background:**

The presence of obstructive sleep apnea (OSA), a novel cardiovascular risk factor, contributes to the development of peripheral arterial diseases (PAD). There is a lack of data showing how often these diseases coexist.

**Aims:**

The aim of the study was to determine the prevalence of OSA in the population of patients with PAD.

**Methods:**

Patients previously qualified for the first revascularization due to PAD were included in the study. All patients underwent an overnight sleep study to detect OSA. Diagnosis of OSA was made when the apnea–hypopnea index (AHI) was ≥5 per hour.

**Results:**

From 141 patients (60% men, age 69.6 ± 9.5 years), OSA was diagnosed in 68 patients (48%). OSA occurred in mild form (5 ≤ AHI < 15/h) in 39 cases (28%), in moderate form (15 ≤ AHI < 30/h) in 21 cases (15%), and in severe form (AHI ≥ 30/h) in 8 cases (6%). Patients without OSA had significantly lower body mass index (BMI; 26.9 ± 5.5 vs. 27.7 ± 5.3 kg/m^2^, *p* = 0.01) and lower hip circumference (97.4 ± 11.7 vs. 98.7 ± 7.4, *p* = 0.04). There were no differences in the distribution of other investigated cardiovascular risk factors and diseases between these groups. There were no significant differences in OSA distribution or its severity between patients with lower extremity artery disease and carotid artery disease.

**Conclusions:**

The prevalence of OSA in patients with PAD is very high, affecting nearly half of the studied population.

## Introduction

Cardiovascular disease (CVD) is a major cause of mortality and disability in European society. The predominant etiology of CVD is atherosclerosis in various anatomical locations. [1] Coronary artery disease (CAD), stroke, and lower extremity artery disease (LEAD) are leading causes of atherosclerotic vascular morbidity from CVD. [2] According to the latest European Society of Cardiology (ESC) guidelines, arterial diseases involving vascular beds other than the aorta and coronary arteries are defined as peripheral arterial diseases (PAD). [3]

Obstructive sleep apnea (OSA) is one of the most common respiratory disorders. [4] OSA is characterized by recurrent episodes of collapse of the upper respiratory tract with persisting respiratory efforts. Its manifestations are apneas or hypopneas followed by desaturation and re-oxygenation cycles, intra-thoracic pressure declines, sympathetic activation, and sleep fragmentation. Patients or bed partners may report snoring or choking and daytime symptoms like sleepiness, fatigue, increased risk for car accidents, and decreased quality of life. [4, 5] OSA with accompanying daytime symptoms is called obstructive sleep apnea syndrome (OSAS). [5]

Evidence from numerous studies on animal models and clinical data suggest a direct and indirect effect of OSA on the development of atherosclerosis, and OSA is proposed as a novel cardiovascular risk factor. [5, 6] OSA and its key mechanism, intermittent hypoxia, may lead to atherogenesis by inducing endothelial dysfunction and inflammation, oxidative stress and lipid peroxidation, dyslipidemia, and insulin resistance. [6] OSA events accompanied by hypoxia and hypercapnia may cause increased sympathetic activity with a substantial increase in heart rate and blood pressure during the resumption of ventilation. [5] In the long term, neural modulation caused by OSA may lead to arterial hypertension, an established CV risk factor. In US and European guidelines, OSA is a recognized modifiable cause of arterial hypertension, affecting approximately 40% of the hypertensive population. [5, 7] Although there is increasing evidence from cohort studies documenting the independent effect of severe OSA on the increased risk of myocardial infarction, stroke, and death from CVD, the frequency of coexistence and the impact of OSA on the occurrence of PAD is still poorly documented. [5]

PAD patients are classified as a population at very high cardiovascular risk. [8] In a large registry trial among all non-cardiac operations, perioperative major adverse cardiovascular and cerebrovascular events occurred most frequently in patients undergoing vascular surgery. [9] The coexistence of OSA and PAD may potentially affect the course of both diseases, worsening the prognosis of patients with PAD and adversely affecting the long-term results of revascularization procedures. Such poor outcomes suggest that patients with PAD warrant routine evaluation not only for classical but also for non-classical cardiovascular risk factors, such as sleep-disordered breathing. The aim of this study was to determine the prevalence of OSA in patients undergoing revascularization for the first time due to PAD and to assess the prevalence of other CVD risk factors in the group of patients with PAD and OSA.

## Materials and methods

### Study design and population

This study is an analysis of data from the PARADISE trial (Peripheral ARtery Atherosclerotic DIsease and SlEep disordered breathing)—an observational cohort study for which the protocol has been previously published. [10] Between 2016 and 2018, all consecutive patients hospitalized in the General and Endocrinological Surgery Clinic of the Medical University of Warsaw, in order to perform revascularization due to PAD (including carotid artery stenting or endarterectomy and stenting, endarterectomy, or lower limb arterial bypass), were evaluated to take part in the trial. In this period of time, 141 patients were enrolled in the study. Inclusion criteria were age 18 to 85 years, previous qualification for the first-ever revascularization due to PAD, and giving written consent. Exclusion criteria were history of revascularization or amputation due to PAD, current use of continuous positive airway pressure, contraindications for a sleep study, disqualification from revascularization, predicted life expectancy less than 6 months (approximated based on age, gender, current medical status, etc.) [11], BMI <18.5 kg/m^2^, and any condition impairing the ability to participate in the trial. All patients included in the study were previously qualified for revascularization by vascular surgeons in accordance with current recommendations and the latest medical knowledge. The presence of PAD was previously confirmed by clinical and imaging studies. All patients on admission underwent a standard examination with an emphasis on the history of CVD and CV risk factors. In addition to standard clinical and biochemical tests, all subjects underwent an overnight sleep study to evaluate possible OSA.

### Sleep study

All enrolled patients, regardless of symptoms suggesting OSA, underwent overnight polygraphy before revascularization intervention. A type 3 portable monitoring device was used in an unattended hospital setting—Embletta® MPR PG (Multi Parameter Recorder—Polygraphy); Flaga, Reykjavik, Iceland. Embletta® meets the American Academy of Sleep Medicine recommendations for evaluation of suspected OSA. [12] Embletta® MPR PG records nasal pressure, thermistor, sound, position, bipolar ECG, thoracic and abdominal effort (respiratory inductance plethysmography—RIP), event button, and pulse oximeter.

### Data analysis

Sleep study recordings were analyzed and scored manually by a study physician trained in sleep medicine. According to the guidelines, apnea was defined as a reduction in airflow >90% compared to baseline lasting at least 10 s. [13] Hypopnea was defined as a transient decrease of airflow in the airways by ≥50% compared to baseline, lasting at least 10 s with a subsequent decrease in blood saturation by ≥4%. This criterion was previously used in a LEAD population by Uriainen et al. [14] In accordance with the AASM recommendations, obstructive, central, and mixed apneas were categorized on the basis of the presence of respiratory effort recorded by abdominal and thoracic RIP belts. Hypopneas were not distinguished. [13] The apnea–hypopnea index (AHI) was defined as the number of obstructive apneas and hypopneas per hour of test time, and diagnosis of OSA was made when the patient was registered with an AHI ≥5 episodes per hour. On the basis of AHI, we categorized patients into four groups: no OSA, AHI <5 per hour; mild OSA, AHI ≥5 and <15 per hour; moderate OSA, AHI ≥15 and <30 per hour; and severe OSA, ≥30 per hour.

### Diagnosis of cardiovascular risk factors and comorbidities

On the basis of clinical history, disease and drug questionnaires, physical examination, body measurements, current blood test, and imaging studies, we determined CV risk factors and comorbidities. All disease entities were defined according to the latest medical knowledge. Selected definitions are presented below:

- Arterial hypertension: repeated blood pressure measurements at least 140/90 mmHg or taking antihypertensive treatment.
- Heart failure (HF): the presence of typical HF signs and symptoms or left ventricular ejection fraction lower than 40%.
- Diabetes: fasting plasma glucose ≥7.0 mmol/L or 2-h plasma glucose during a 75-g oral glucose tolerance test ≥11.1 mmol/L or random plasma glucose ≥11.1 mmol/L with hyperglycemia symptoms or taking antidiabetic treatment.
- Chronic kidney disease: glomerular filtration rate <60 mL/min/1.73 m^2^ measured for at least 3 months.
- Positive family history: CAD or other atherosclerotic diseases occurring in men before 55 years of life or in women before 65 years of life.
- Previous myocardial infarction, stroke, asthma, chronic obstructive pulmonary disease: on the basis of medical records.
- Body weight categories were determined on the basis of BMI: normal, ≥18.5 and <25 kg/m^2^; overweight, ≥25 and <30 kg/m^2^; and obesity, ≥30 kg/m^2^.

### Ethical and legal issues

The study protocol including questionnaires, clinical examinations, body measurements, blood tests, polygraphy studies, as well as patient information and informed consent were approved by the Bioethics Commission by the Medical University of Warsaw (KB/196/2015). Each patient was assigned a unique identification number for the protection of personal information. Researchers, statisticians, and controlling bodies were blinded to research data. No third parties had access to the research data.

### Statistical analysis

Patients were characterized using standard statistical tools; for continuous parameters, mean values and standard deviations or medians were used. Parameters with discreet character were presented in frequency tables. Normality of distribution was determined on the basis of D’Agostino–Pearson and Kolmogorov–Smirnov tests. To compare mean values between groups, Student’s *t* test was used for data with normal distribution and Mann–Whitney *U* test was used for data deviating from a normal distribution. For frequency comparisons, depending on the sample size, *χ*^2^ test or non-parametric Fisher’s exact test was used. For comparison of mean values between more than two independent groups, depending on the distribution, analysis of variance (ANOVA) or the Kruskal–Wallis test was used. All statistical calculations were made using SPSS v.21.0 software (SPSS Inc., Chicago, IL, USA).

## Results

**Figure 1:**
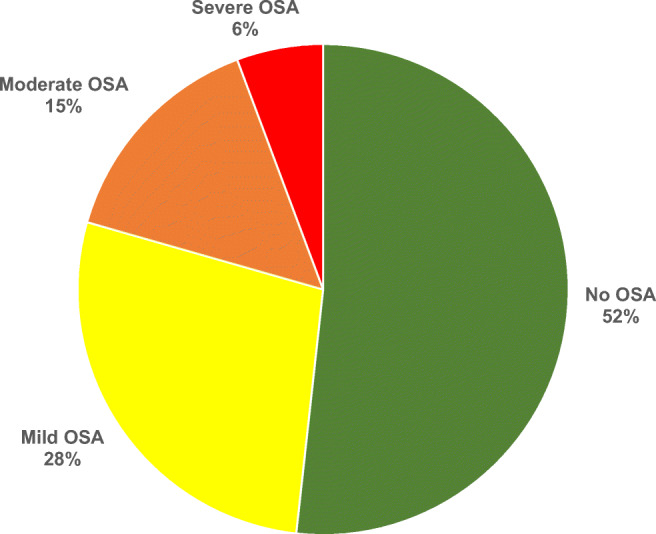
Prevalence of obstructive sleep apnea in patients with peripheral arterial disease

Of 141 consecutive patients enrolled, the majority were men (60%) at mean age of 69.6 ± 9.5 years. A majority of patients (102, 72.3%) were qualified for revascularization due to LEAD. The rest of the study population (39, 28%) was referred for carotid artery revascularization. OSA was diagnosed in 68 patients (48%), mild in 39 cases (28%), moderate in 21 (15%), and severe in 8 (6%) (Fig. 1). Demographic and clinical characteristics of the patients are presented in Table 1.

**Table 1:** Demographic and clinical characteristics of all patients (*n* = 141)

Parameter	Value (mean ± SD or *n* (%))
Age (years)	69.6 ± 9.5
Male	84 (59.6%)
LEAD	102 (72.3%)
Carotid artery disease	39 (27.7%)
BMI (kg/m^2^)	27.3 ± 5.4
Normal body weight	50 (35.5%)
Overweight	58 (41.1%)
Obesity	33 (23.4%)
Neck circumference (cm)	40.5 ± 14.3
Chest circumference (cm)	98.2 ± 10.9
Waist circumference (cm)	97.3 ± 11.4
Hip circumference (cm)	98.0 ± 9.8
Arm circumference (cm)	29.1 ± 2.9
Arterial hypertension	104 (73.8%)
Previous myocardial infarction	28 (19.9%)
Previous stroke	23 (16.3%)
Heart failure	17 (12.1%)
Diabetes mellitus	47 (33.3%)
Chronic kidney disease	14 (9.9%)
Asthma	3 (2.1%)
COPD	8 (5.7%)
Positive family history	43 (30.5%)
Smoker	109 (77.3%)
Total cholesterol mmol·L^−1^	4.84 ± 2.58
LDL mmol·L^−1^	2.60 ± 1.09
HDL mmol·L^−1^	1.31 ± 0.37
TGL mmol·L^−1^	1.53 ± 0.72
Statin use	46 (32.6%)
CRP mg·L^−1^	10.9 ± 29.4
Data related to obstructive sleep apnea (OSA)	
AHI (events/h)	8.6 ± 10.2
OSA (total)	68 (48.2%)
Mild OSA	39 (27.7%)
Moderate OSA	21 (14.9%)
Severe OSA	8 (5.7%)

*AHI* apnea–hypopnea index, *BMI* body mass index, *COPD* chronic obstructive pulmonary disease, *CRP* C-reactive protein, *HDL* high-density lipoprotein, *LDL* low-density lipoprotein, *LEAD* lower extremity artery disease, *TGL* triglycerides

The study population was analyzed after division into two subgroups: patients operated on due to carotid artery disease and patients operated on due to LEAD. Detailed data for these patients are presented in Table 2. We observed substantially higher ischemic stroke prevalence in the carotid artery disease group when compared to the LEAD group (41% vs. 7%, *p* < 0.0001). We also observed a trend toward a more common presence of heart failure in the LEAD group. Compared to LEAD patients, patients with carotid artery disease had statistically lower LDL-C levels (2.02 ± 0.78 vs. 2.82 ± 1.11 mmol/L, *p* = 0.04) and CRP levels (4.2 ± 6.6 vs. 13.4 ± 33.9 mg/L, *p* = 0.007). In anthropometric measurements, the only significant difference was in arm circumference. In tested subgroups, no statistically significant differences were found in the distribution of other CV diseases or CV risk factors, including OSA prevalence or OSA severity (see Table 2).

**Table 2:** Demographic and clinical characteristics—comparison of subgroups separated on the basis of operated localization

Parameter	Patients with carotid artery disease (*n* = 39) Value (mean ± SD or *n* (%))	Patients with LEAD (*n* = 102) Value (mean ± SD or *n* (%))	*p* value
Age (years)	70.1 ± 10.5	69.4 ± 9.1	0.51
Male	24 (61.5%)	60 (58.8%)	0.46
BMI (kg/m^2^)	27.6 ± 4.8	27.2 ± 5.6	0.93
Normal body weight	14 (35.9%)	36 (35.3%)	0.64
Overweight	14 (35.9%)	44 (43.1%)	0.64
Obesity	11 (28.2%)	22 (21.6%)	0.64
Neck circumference (cm)	39.4 ± 3.5	40.8 ± 15.5	0.40
Chest circumference (cm)	100.5 ± 9.0	97.4 ± 11.4	0.48
Waist circumference (cm)	98.9 ± 12.0	96.7 ± 11.5	0.76
Hip circumference (cm)	97.5 ± 9.9	98.2 ± 9.9	0.58
Arm circumference (cm)	29.5 ± 3.9	29.0 ± 3.1	0.03
Arterial hypertension	32 (82.1%)	72 (70.6%)	0.12
Previous myocardial infarction	6 (15.4%)	22 (21.6%)	0.28
Previous stroke	16 (41.0%)	7 (6.9%)	<0.0001
Heart failure	2 (5.1%)	15 (14.7%)	0.09
Diabetes mellitus	15 (38.5%)	32 (31.4%)	0.27
Chronic Kidney disease	5 (12.8%)	9 (8.8%)	0.33
Asthma	1 (2.6%)	2 (1.9%)	0.87
COPD	3 (7.7%)	5 (49%)	0.98
Positive family history	11 (28.2%)	32 (31.4%)	0.44
Smoker	29 (74.4%)	80 (78.4%)	0.38
Total cholesterol mmol·L^−1^	3.96 ± 1.06	5.17 ± 2.90	0.25
LDL mmol·L^−1^	2.02 ± 0.78	2.82 ± 1.11	0.04
HDL mmol·L^−1^	1.25 ± 0.35	1.34 ± 0.37	0.52
TGL mmol·L^−1^	1.52 ± 0.82	1.53 ± 0.69	0.08
Statin use	14 (35.9%)	32 (31.4%)	0.37
CRP mg·L^−1^	4.2 ± 6.6	13.4 ± 33.9	0.007
Data related to obstructive sleep apnea (OSA)			
AHI (events/h)	9.4 ± 10.9	8.2 ± 10	0.60
OSA (total)	19 (48.7%)	49 (48.0%)	0.55
Mild OSA	11 (28.2%)	28 (27.5%)	0.97
Moderate OSA	6 (15.4%)	15 (14.7%)	0.97
Severe OSA	2 (5.1%)	6 (5.9%)	0.97

*AHI* apnea–hypopnea index, *BMI* body mass index, *COPD* chronic obstructive pulmonary disease, *CRP* C-reactive protein, *HDL* high-density lipoprotein, *LDL* low-density lipoprotein, *LEAD* lower extremity artery disease, TGL triglycerides

Analysis of study population subgroups separated on the basis of presence of OSA is presented in Table 3. In these subgroups, there were no statistically important differences in distribution of CV diseases or in the majority of CV risk factors. However, compared to patients with OSA, those without OSA had, respectively, significantly lower BMI (26.9 ± 5.5 vs. 27.7 ± 5.3, *p* = 0.01), significantly lower hip circumference (97.4 ± 11.7 vs. 98.7 ± 7.4, *p* = 0.04), more common normal body weight (29, 40% vs. 21, 31%, *p* = 0.04), higher total cholesterol (5.09 ± 3.41 vs. 4.56 ± 1.05, *p* = 0.04), and were less often treated with statins (19% vs. 47%, *p* < 0.0001).

**Table 3:** Demographic and clinical characteristics—comparison of subgroups separated on the basis of presence of obstructive sleep apnea (OSA)

Parameter	Patients without OSA (*n* = 73) Value (mean ± SD or *n* (%))	Patients with OSA (*n* = 68) Value (mean ± SD or *n* (%))	*p* value
Age (years)	69.6 ± 8.8	69.6 ± 10.2	0.35
Male	40 (54.8%)	44 (64.7%)	0.15
LEAD	53 (72.6%)	49 (72.1%)	0.55
Carotid artery disease	20 (27.4%)	19 (27.9%)	0.55
BMI (kg/m^2^)	26.9 ± 5.5	27.7 ± 5.3	0.01
Normal body weight	29 (39.7%)	21 (30.9%)	0.04
Overweight	29 (39.7%)	29 (42.6%)	0.18
Obesity	15 (20.5%)	18 (26.5%)	0.08
Neck circumference (cm)	39.4 ± 6.9	41.7 ± 18.4	0.43
Chest circumference (cm)	98.6 ± 9.5	97.8 ± 12.3	0.48
Waist circumference (cm)	96.9 ± 11.9	97.7 ± 11.5	0.97
Hip circumference (cm)	97.4 ± 11.7	98.7 ± 7.4	0.04
Arm circumference (cm)	29.4 ± 3.6	28.8 ± 2.9	0.30
Total cholesterol mmol·L^−1^	5.09 ± 3.41	4.56 ± 1.05	0.04
LDL mmol·L^−1^	2.70 ± 1.19	2.49 ± 0.97	0.19
HDL mmol·L^−1^	1.27 ± 0.36	1.36 ± 0.38	0.14
TGL mmol·L^−1^	1.57 ± 0.78	1.48 ± 0.66	0.47
Statin use	14(19.2%)	32 (47.1%)	<0.0001
CRP mg·L^−1^	11.7 ± 29.7	9.9 ± 29.2	0.47
Arterial hypertension	55 (75.3%)	49 (72.1%)	0.40
Previous myocardial infarction	14 (19.2%)	14 (20.6%)	0.51
Previous stroke	11 (15.1%)	12 (17.6%)	0.43
Heart failure	8 (11.0%)	9 (13.2%)	0.44
Diabetes mellitus	25 (34.2%)	22 (32.4%)	0.48
Chronic kidney disease	10 (13.7%)	4 (5.9%)	0.11
Asthma	2 (2.7%)	1 (1.5%)	0.68
COPD	5 (6.8%)	3 (4.4%)	0.32
Positive family history	22 (30.1%)	21 (30.9%)	0.54
Smoking	59 (80.8%)	50 (73.5%)	0.21

*AHI* apnea–hypopnea index, *BMI* body mass index, *COPD* chronic obstructive pulmonary disease, *CRP* C-reactive protein, *HDL* high-density lipoprotein, *LDL* low-density lipoprotein, *LEAD* lower extremity artery disease, *TGL* triglycerides

## Discussion

This study constitutes the largest cohort of patients with PAD tested for the presence of OSA. This is the first study including both LEAD and carotid artery disease populations. The main finding of the trial was a high prevalence of OSA in patients with severe PAD in the Polish population. This prevalence substantially exceeds that previously reported in the general population. For example, in the Wisconsin Sleep Cohort Study, the prevalence of sleep-disordered breathing defined as AHI ≥5/h was estimated at 9% of women and 24% of men between 30 and 60 years old. [15] In our study, OSA affected nearly half (48%) of the tested cohort, though in the majority of cases, the disease was mild (28%) or moderate (15%). These findings differ substantially from previously published data. Utriainen et al. reported OSA prevalence of 85% in a population with severe LEAD (*n* = 82), of which 28% cases were mild, 28% moderate, and 29% severe. [14] Schahab et al. reported OSA prevalence of 82% in a population with LEAD (*n* = 59), of which 35% were mild, 27% moderate, and 38% severe. [16] These differences in distribution cannot be explained by the localization of vascular lesions in tested cohorts, as data from the current study revealed no significant differences in OSA prevalence or its severity between LEAD and carotid artery disease subgroups. It is worth noting that Utriainen et al. had the same scoring criteria for AHI assessment applied in the current study while Schahab et al. used a more liberal criterion for hypopnea scoring, i.e., the same criterion for a reduction in airflow (≥50%) with smaller oxygen desaturation range (≥3%). [14, 16] Reasons for distribution dissimilarities may lie in different inclusion/exclusion criteria or bias associated with testing of small cohorts.

As expected, the prevalence of other risk factors for CV diseases is very high in patients with PAD exceeding that reported in the general population. Two of the most common risk factors are hypertension and tobacco use, which affect about three fourths of patients with PAD undergoing primary arterial revascularization. Also, a high prevalence of diabetes and a family history of cardiovascular diseases contribute negatively to the risk profile of these patients. It is important to note that despite high prevalence of CV risk factors in this population with atherosclerosis, recommended statin treatment was prescribed for only one third. Improvement in the application of pharmacotherapy prior to surgery may potentially contribute to postponing treatment, improving quality of life as well as prognosis in this group of patients.

Obesity is the most frequently documented risk factor for the development of OSA. In population with BMI >40 kg/m^2^, prevalence of OSA has been reported between 40% and 90%. [17] This observed relationship is proposed to be caused by anatomical and neuro-muscular mechanisms. [17] Similar to the general population, OSA was more common in our patients with increased body weight and with higher values of anthropometric measurements. On the other hand, in our cohort, the majority of patients with OSA were not obese, consistent with previous observations in population with severe LEAD. [14] The prevalence of OSA is substantially higher in men than women in the general population, this proportion varying from 2:1 to 4:1. [18] In our cohort, there was a non-significant trend toward a higher percentage of men in the subgroup with OSA.

Our study has important limitations. The cross-sectional design of the trial makes it impossible to establish a causal relationship. Our liberal exclusion policy, on the one hand, gives an opportunity to assess prevalence in a real life population with PAD, while on the other hand, the heterogeneous population with many existing comorbidities may overestimate the prevalence of OSA in populations with isolated PAD without serious comorbidities.

We conclude that unrecognized OSA is highly prevalent in patients with both severe lower extremity artery disease and severe carotid artery disease. However, in most cases, the OSA is mild to moderate in severity. Because diagnosis and treatment of OSA may potentially affect outcomes in this population at very high cardiovascular risk, it is important to maintain a high index of suspicion for OSA and to assess these patients with sleep studies.
